# Total Extirpation of the Stomach

**Published:** 1898-07-23

**Authors:** 


### TOTAL EXTIRPATION OF THE STOMACH.

It would, no doubt, be wrong to say that in the construction of man the provision of gastric juice was an afterthought; nevertheless, the success which has again followed the total extirpation of the stomach by the operation of œsophago-enterostomy shows what, indeed, has already been proved by experiments upon animals —that gastric digestion is not an essential part of the digestive process. Owing to the fact that the eating of food is not a continuous process, most animals are provided with pouches at certain points in the digestive canal; and, perhaps, we may look at the stomach as in essence a bag in which the bulk of a meal may be retained for such time as may enable the food to pass through the rest of the canal in a steady and more or less even flow. With the wonderful adaptiveness which, to use the old-fashioned phraseology, we see in "Nature" on every side, this retardation and accumulation of food in the stomach is taken advantage of to advance it one stage in its progress towards complete digestion. This, however, we may take to be non-essential, the primary object of the stomach being that it may act as a receptacle for food rather than as an organ of digestion.

